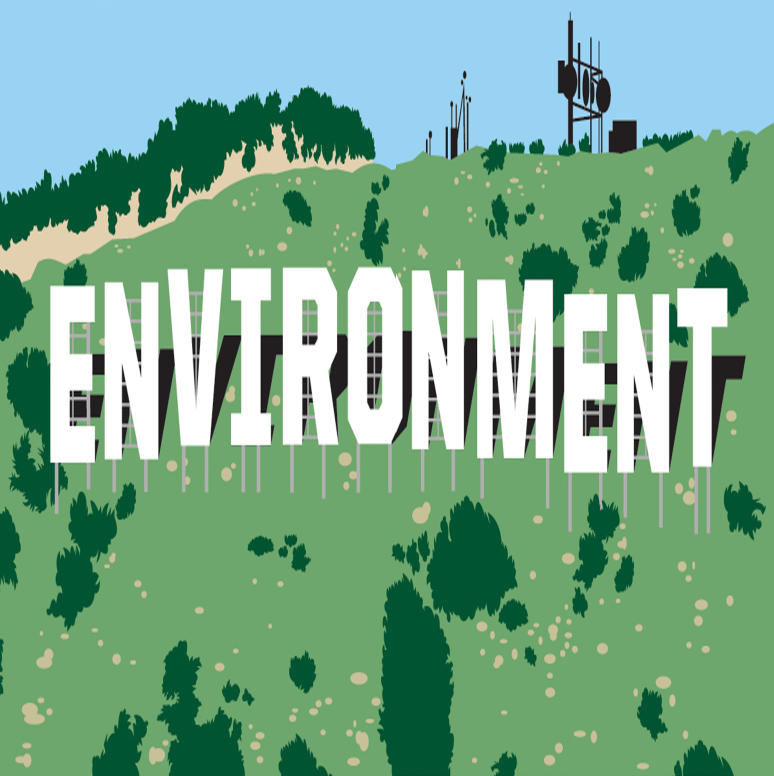# ENVIRONMENT: California Out in Front

**DOI:** 10.1289/ehp.115-a144

**Published:** 2007-03

**Authors:** Charles W. Schmidt

When it comes to ecological diversity, California has it all: snow-capped mountains, wide deserts, scenic beaches, and some of the worst environmental problems in the country. Six of the country’s ten most polluted cities—Los Angeles, Bakersfield, Fresno–Madera, Visalia–Porterville, Merced, and Sacramento—are found in California, where children face fivefold greater risks of reduced lung function compared with children who live in less-polluted areas. Beyond its air pollution problems, California could also face catastrophic consequences from climate change. Assuming warming trends continue at their present rates, experts generally agree that the Sierra snowpack—which is crucial to the state’s drinking water supply—could decline by 50–90% by the century’s end.

With statistics like that, environmentalism has become a powerful force in California. According to a 2006 survey conducted by the Public Policy Institute of California (PPIC), a San Francisco–based research organization, 65% of Californians don’t think the federal government is doing enough to combat global warming. Two-thirds of the population support state efforts to address climate change, while an equal number support tougher air pollution standards on new vehicles, even if it makes vehicles more expensive.

California legislators have responded with some of the strongest environmental laws ever passed. Whereas the U.S. government has yet to regulate carbon dioxide, California recently passed AB 32, a groundbreaking law signed by governor Arnold Schwarzenegger in September 2006 that directs industries to reduce all greenhouse gas emissions by 25% over the next 13 years. Another law—AB 1493, which was enacted in 2002—directs automakers to reduce greenhouse gases emitted by passenger vehicles sold in California after 2009, with a 30% reduction in statewide vehicular emissions by 2016. (That law is currently being challenged by a lawsuit from the automotive industry.)

This year, California will consider a statewide green chemistry policy that could exceed the scope of the federal Toxic Substances Control Act (TSCA), which sets national policy on chemicals used in products and industrial processes. Local governments have also tightened environmental controls. San Francisco, for instance, recently passed the country’s first ban on baby products containing bisphenol A and has also regulated levels of phthalates in these products. Bisphenol A and phthalates are both suspected endocrine disruptors.

Coming from one of the world’s largest economies, these preemptive legislative efforts have impressive clout. “California provides an example [for other states],” says Cympie Payne, associate director of the California Center for Law and Policy at the University of California (UC), Berkeley. “Other states find it easier to model their own laws on those that another state has already put into effect.”

## Clearing the Air

California’s aggressive environmental policies build on a long history. In 1965, the state became the first to regulate vehicle exhaust by setting limits on hydrocarbons and carbon monoxide emissions. Two years later, the newly formed California Air Resources Board (ARB)—now part of the California EPA—set the nation’s first air quality standards for total suspended particulates, photochemical oxidants, sulfur dioxide, nitrogen dioxide, and other pollutants.

Early on, U.S. lawmakers recognized that California had a terrible problem with air pollution. Living in low-density sprawl, Southern Californians travel everywhere by car, generating exhaust plumes that get trapped at ground level in the area’s low-lying valleys. Truck traffic across the Mexican border, in addition to emissions from the Los Angeles–Long Beach port complex—the largest man-made harbor in the western United States—also contribute to the region’s poor air quality.

To give the state more leverage on pollution control, Congress allowed California to enforce pollution standards that might be more stringent than those passed by the federal government. That allowance was first introduced in the Federal Air Quality Act of 1967, and later codified in Section 209 amendments to the Clean Air Act (CAA). California has since set the nation’s tightest standards for ozone and particulate matter, according to ARB spokesman Jerry Martin. Other states, meanwhile, have no comparable authority when it comes to devising their own air quality standards. Rather, the CAA allows them to choose whether to adopt federal standards or the more stringent California standards.

If California’s strict environmental policies were triggered by traditional air pollution, its current reputation as a green pioneer has more to do with recent initiatives on climate change. By signing AB 1493, governor Gray Davis put California at the leading edge of government efforts to regulate greenhouse gases. Reflecting California’s legislative influence, ten other states—New York, Massachusetts, Connecticut, Maryland, Delaware, Rhode Island, Maine, Vermont, Washington, and Oregon—along with Canada have all adopted the same goal.

But AB 1493 has its detractors, particularly among the auto industry. The stakes are huge for U.S. automakers: California accounts for 10% of their total sales. Auto industry lobbyists have overcome every congressional attempt to improve fuel efficiency standards since 1990. But Martin stresses that although better fuel efficiency does advance AB 1493’s goals, automakers have other alternatives for reducing emissions, such as cutting back on the use of halogenated refrigerants, which exceed carbon dioxide in terms of greenhouse potency. Automakers can also sell more “flex-fuel” vehicles that run on ethanol blends, he says.

According to Martin, this flexibility in options distinguishes AB 1493 from corporate average fuel economy (CAFE) standards, which dictate only the minimum average miles per gallon that cars of a particular class need to achieve. “Our standards are for greenhouse gases; we call them global warming standards,” Martin says. “And they include not just carbon dioxide but other gases like methane and [halogenated refrigerants].”

But the auto industry sees things differently. Charlie Territo, a spokesman for the Alliance of Automobile Manufacturers, a national industry trade group, calls AB 1493 a thinly veiled attempt to regulate fuel economy. Moreover, he adds, California has no authority to impose higher fuel economy standards because its special state status on the environment applies only to the CAA. CAFE standards, on the other hand, are administered by the National Energy Policy and Conservation Act, under which California has no special status. Equally significant, California can set its own state standards only for criteria pollutants listed under the CAA, a list that doesn’t yet include carbon dioxide, he says.

The U.S. Supreme Court will determine later this year if the U.S. EPA must regulate carbon dioxide as an air pollutant. At issue is *Massachusetts et al. v. Environmental Protection Agency et al.*, wherein Massachusetts represents a coalition of stakeholders who believe carbon dioxide should be regulated to limit global warming. The U.S. EPA doesn’t want to regulate carbon dioxide without better knowledge of the gas’s role in climate change, according to James R. Milkey, the counsel of record for the Supreme Court case. Meanwhile, the Alliance of Automobile Manufacturers and other auto industry groups have sued California, Rhode Island, and Vermont, arguing that the application of AB 1493 (and equivalent counterparts in other states) is illegal. The suit, originally scheduled for trial in California beginning 31 January 2007, has been postponed until the spring pending the Supreme Court’s decision.

## Meeting Legislative Goals

The upcoming Supreme Court decision could also be critical for AB 32’s goal of reducing California’s total greenhouse gas emissions to 25% below 1990 levels by 2020. A press release issued by California’s Office of the Governor on 27 September 2006 stated that AB 32 is a “landmark bill that establishes a first-in-the-world, comprehensive program of regulatory and market mechanisms to achieve real, quantifiable, cost-effective reductions of greenhouse gases.” Says Martin, “AB 1493 just focuses on cars, but AB 32 covers everything that uses energy in some way. And since California is the twelfth largest producer of greenhouse gases in the world, that’s a big deal.”

The law directs the ARB to determine how California can meet its emissions reduction goal. Toward that end, the board will develop appropriate regulations (which it will also enforce) and create a reporting system to track and monitor greenhouse gas emissions. With ARB approval, the law could allow California industries to trade emissions on global markets. That effort would apply economic forces to the goal of limiting emissions below a statewide cap yet to be identified, which will be phased in starting in 2012.

As a first priority, Martin says the agency is producing an emissions inventory, to quantify how much carbon dioxide California industries and their suppliers produce. At the same time, the ARB is compiling a list of “discrete early actions”—simple measures—to limit greenhouse gases that can be phased in by 2010. Along those lines, Schwarzenegger recently ordered that the carbon content of all transportation-related fuels burned in California must be reduced by 10% by 2020. The ARB is currently reviewing the governor’s order to see if it qualifies as a discrete early action under AB 32. Martin suggests that it might, and adds that fuel companies could meet the mandate in a number of ways, for instance, by selling more biofuels.

In contrast to AB 1493, lawsuits against AB 32 haven’t been filed. That’s because specific measures targeting individual industries aren’t yet known, Martin explains. Once those measures are identified, affected industries will sue accordingly, he predicts.

Meanwhile, stakeholders everywhere are anxious to know if California can limit greenhouse gases without wrecking its economy. “It’s going to be tricky,” concedes Dominic DiMare, vice president of government relations at the California Chamber of Commerce, which opposed both AB 1493 and AB 32. “The law could harm the economy, but it could also help it, and that depends on how it’s implemented, which is something we still don’t know. My concern is that some companies might leave California rather than face those restrictions. Then they might wind up in other areas where they pollute even more than they do here.”

Countering those concerns, UC Berkeley adjunct professor David Roland-Holst was quoted in the 15 September 2006 *New York Times* as estimating that AB 32 could pump $60 billion and 17,000 jobs into the California economy by 2020, by attracting investment in alternative energy. Roland-Holst could not be reached for comment.

## Green Chemistry

Apart from global warming, California’s next big effort on the environment could come from a burgeoning green chemistry policy—that is, one that identifies safer chemicals and processes. That pending effort responds to a 2006 UC Berkeley report titled *Green Chemistry in California: A Framework for Leadership in Chemicals Policy and Innovation*, which concluded that federal policies under TSCA don’t do enough to protect public health. The 130-page document was drafted at the request of the California legislature.

According to lead author Michael P. Wilson, a research scientist at the UC Berkeley Center for Occupational and Environmental Health, TSCA’s data requirements impede the transparency and oversight that are necessary to protect public health and allow proper function of the chemicals market. TSCA does not require producers to generate data on chemical toxicity, he says, and that produces uncertainty for companies that purchase chemicals. Moreover, he says, TSCA constrains the government’s ability to control the sale of hazardous chemicals, which allows these substances to remain competitive in the market. The report concluded that these market conditions have dampened interest by industry in green chemistry.

“We have a failure in the U.S. chemicals market,” Wilson stresses. “Chemicals are marketed on the basis of their function, price, and performance, but the hazard piece is still largely missing.”

Responding to the report’s message, state senator Joe Simitian, who chairs the California Senate Environmental Quality Committee, is investigating options for a new green chemistry policy that might address TSCA’s shortcomings. Bruce Jennings, a senior advisor to the California legislature, with whom Simitian collaborates, says a number of green chemistry bills could go to the floor this year. One would create a clearinghouse on alternatives to hazardous chemicals, geared toward small companies that lack access to that type of information and patterned after a similar U.S. EPA program, Design for the Environment. Another would require the producers of high production volume chemicals to submit environmental health information to California, in addition to information about the use and disposal of such chemicals.

Simitian was quoted in the 2 November 2006 *Capitol Weekly* as saying that he wants to apply a precautionary approach to California’s emerging chemical regulations. That approach—popular among European Union countries—shifts the burden of proof regarding chemical safety to manufacturers instead of regulators. The precautionary principle, as it is often called, drives some of the European Union’s most sweeping—and controversial—environmental initiatives, particularly the REACH (Registration, Evaluation, and Authorisation of Chemicals) directive, which requires that chemicals manufactured or imported at volumes of greater than one metric ton be registered with the European Chemicals Agency. Under the REACH initiative, which goes into effect in June 2007, some toxic chemicals could be phased out in favor of less toxic alternatives.

U.S. industries have fought against REACH, which will affect their exports to Europe. Now some industry stakeholders worry that California’s potential green chemistry policies could be a stepping stone toward REACH implementation in the United States. “We’re concerned this could impose added costs on California businesses,” says John Ulrich, executive director of the Chemical Industry Council of California, a trade group. “Anything that increases the cost of manufacturing across the board in California will discourage manufacturing here. I’m afraid a legislative package that claims to be green chemistry will go down a conventional route of legislating based on unfounded science, using timetables that aren’t credible or achievable.”

While claiming it’s still too early to know what form the policy will take, Jennings stresses the goal isn’t to replicate REACH or any other European initiative. “We want to complement what they’re doing,” he says. “And there are plenty of industry players who face challenges with operating in global markets when they lack information about the chemical content of their products. Chemical producers may be troubled by changes in the law, but we think downstream users will welcome efforts to give them more information.”

In support of that view, Rachelle Reyes Wenger, who manages public policy and advocacy at Catholic Healthcare West, a San Francisco–based company that owns 42 hospitals and employs 44,000 people, says better information on chemical safety and alternative products can be good for business. She notes that her company recently awarded a multiyear $70 million contract to a company that supplies intravenous bags that do not contain polyvinyl chloride, phthalates, or other toxic chemicals. “With our purchasing power, we can really make a difference,” she says. “A comprehensive chemical policy could hurt finances initially, but not in the long run. It’s ultimately better not just for the financial bottom line, but also for the moral bottom line.”

And of course, for the environmental bottom line. California’s lawmakers have apparently decided that sacrifices made now to achieve environmental goals are worth the future benefits, not just for health and ecology, but for the long-term sustainability of the state’s industries. Ultimately, California’s paving a road forward on which others may inevitably follow.

## Figures and Tables

**Figure:**